# Supplementary material for: Comparison of the Response to the CXCR4 Antagonist AMD3100 during the Development of Retinal Organoids Derived from ES Cells and Zebrafish Retina
Source: Int J Mol Sci. 2022 Jun 25;23(13):7088. doi: 10.3390/ijms23137088 (PMC9266567; doi:10.3390/ijms23137088)
Supplement: Supplementary file 1 [file ijms-23-07088-s001.zip › ijms-1725824-supplementary.pdf]

Supplementary Materials:

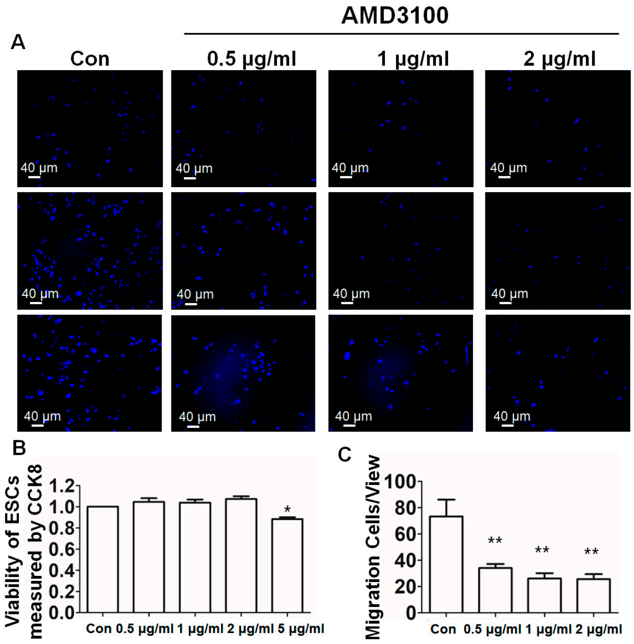

Figure S1. The concentration of AMD3100 was confirmed by Transwell Cell Migration Assay and CCK8. (**A**) Transwell Cell Migration Assay was conducted in hESCs cultured in mTesr medium, with or without 0.5 µg/mL, 1 µg/mL, or 2 µg/mL AMD3100 (* $p < 0.05$). (**B**) ImageJ quantified the number of migrating cells. (**C**) CCK8 Assay was conducted in hESCs cultured in mTesr medium, with or without 0.5 µg/mL, 1 µg/mL, 2 µg/mL, or 5µg/mL AMD3100 (** $p < 0.01$). Scale bars: 40 µm.

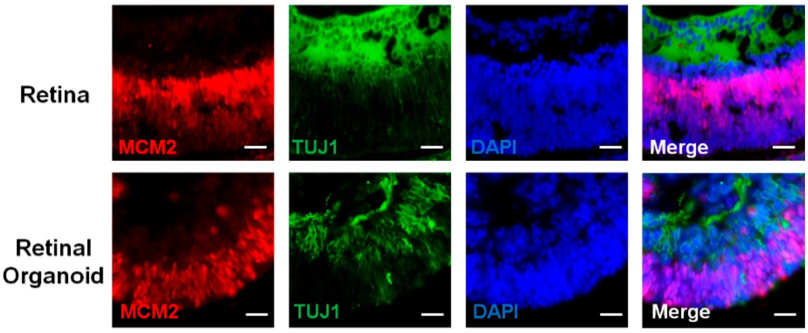

Figure S2. The structure of retinal organoid and mouse (postnatal day 1) retina tissue in vivo. Retinal progenitor cells were stained by MCM2 (red) and retinal neurons were stained by TUJ1 (green). In retina organoid and retinal tissue, the progenitor cells located in INBL and the neurons were mostly located in the ganglion cells layer. Scale bars: 50 µm.

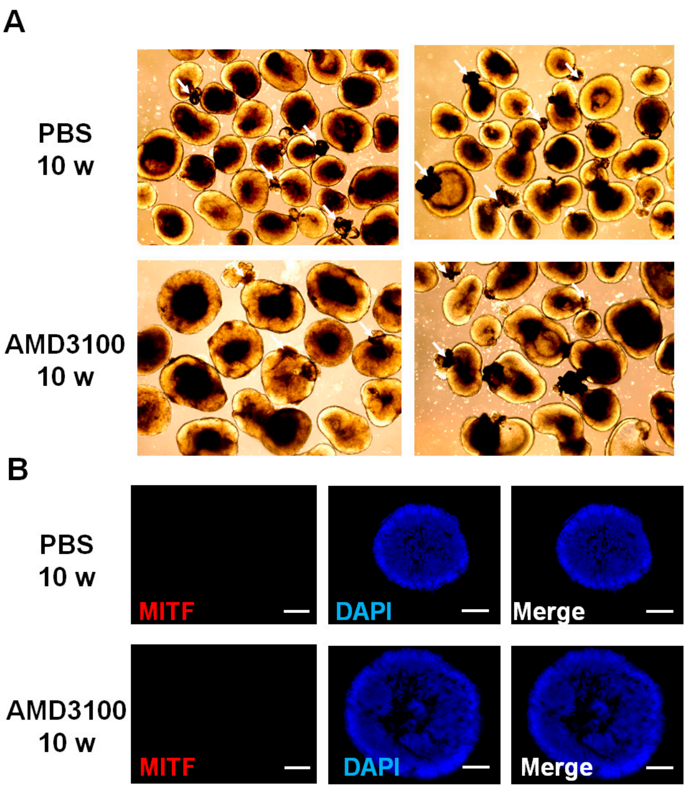

Figure S3. The retinal pigment epithelium (RPE) tissue in retina organoids. (**A**) Images of retinal organoids in the PBS and AMD3100 treatment group. The neural retina was continuous with the adjacent RPE (white arrow). (**B**) The RPE tissue was stained by MITF in the neural retina. Scale bars: 100 µm.

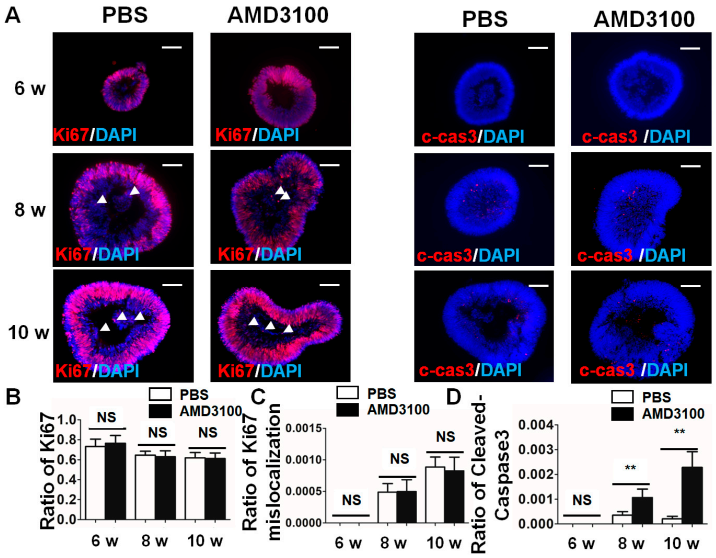

Figure S4. CXCR4 blockage does not effect cell proliferation but causes cell apoptosis. (**A**) Retinal proliferative cells were stained by Ki67 and apoptosis cells were stained by Cleaved-Caspase 3. (**B**) The relative ratio of Ki67-positive cells to all cells (nuclei were stained with DAPI) was not significantly different between the two groups (NS: Not Significant, $p > 0.1$, n = 10). (**C**) The relative ratio of Ki67-positive cells mislocalization (not located in the INBL, white arrow) to all cells (nuclei were stained with DAPI) was not significantly different between the two groups (NS: Not Significant, $p > 0.1$, n = 10). (**D**) The relative ratio of Cleaved-Caspase 3-positive cells to all cells (nuclei were stained with DAPI) was

significantly increased in the AMD3100 group (** $p < 0.01$, NS: Not Significant, $p > 0.1$, n = 10). Scale bars: 100 μm.

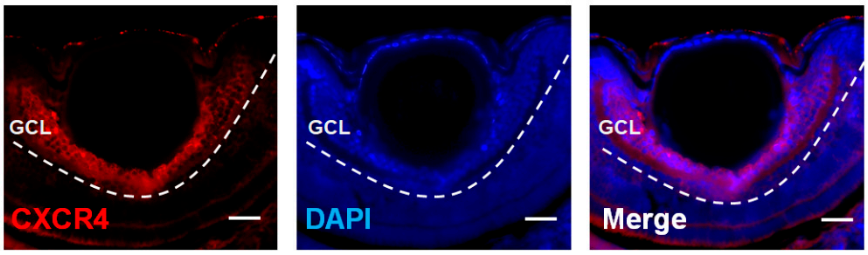

Figure S5. The CXCR4 expression in 72 hpf zebrafish retina. CXCR4 expresses mostly in the 72 hpf ganglion cells. Scale bars: 100 μm.
